# EnHERV: Enrichment analysis of specific human endogenous retrovirus patterns and their neighboring genes

**DOI:** 10.1371/journal.pone.0177119

**Published:** 2017-05-04

**Authors:** Pumipat Tongyoo, Yingyos Avihingsanon, Santhitham Prom-On, Apiwat Mutirangura, Wuttichai Mhuantong, Nattiya Hirankarn

**Affiliations:** 1 Inter-Department Program of Biomedical Sciences, Faculty of Graduate School, Chulalongkorn University, Bangkok, Thailand; 2 Center of Excellence in Immunology and Immune Mediated Diseases, Faculty of Medicine, Chulalongkorn University, Bangkok, Thailand; 3 Division of Nephrology, Department of Medicine, Chulalongkorn University and King Chulalongkorn Memorial Hospital, Bangkok, Thailand; 4 Computer Engineering Department, Faculty of Engineering, King Mongkut's University of Technology Thonburi, Bangmod, Thungkhru, Bangkok, Thailand; 5 Center of Excellence of Molecular Genetics of Cancer and Human Diseases, Department of Anatomy, Faculty of Medicine, Chulalongkorn University, Bangkok, Thailand; 6 Enzyme Technology Laboratory, Microbial Biotechnology and Biochemicals Research Unit, National Center for Genetic Engineering and Biotechnology (BIOTEC), Khlong Luang, Pathum Thani; 7 Department of Microbiology, Faculty of Medicine, Chulalongkorn University, Bangkok, Thailand; Plymouth University, UNITED KINGDOM

## Abstract

Human endogenous retroviruses (HERVs) are flanked by long terminal repeats (LTRs), which contain the regulation part of the retrovirus. Remaining HERVs constitute 7% to 8% of the present day human genome, and most have been identified as solo LTRs. The HERV sequences have been associated with several molecular functions as well as certain diseases in human, but their roles in human diseases are yet to be established. We designed EnHERV to make accessible the identified endogenous retrovirus repetitive sequences from Repbase Update (a database of eukaryotic repetitive elements) that are present in the human genome. Defragmentation process was done to improve the RepeatMasker annotation output. The defragmented elements were used as core database in EnHERV. EnHERV is available at http://sysbio.chula.ac.th/enherv and can be searched using either gene lists of user interest or HERV characteristics. Besides the search function, EnHERV also provides an enrichment analysis function that allows users to perform enrichment analysis between selected HERV characteristics and user-input gene lists, especially genes with the expression profile of a certain disease. EnHERV will facilitate exploratory studies of specific HERV characteristics that control gene expression patterns related to various disease conditions. Here we analyzed 25 selected HERV groups/names from all four HERV superfamilies, using the sense and anti-sense directions of the HERV and gene expression profiles from 49 specific tissue and disease conditions. We found that intragenic HERVs were associated with down-regulated genes in most cancer conditions and in psoriatic skin tissues and associated with up-regulated genes in immune cells particularly from systemic lupus erythematosus (SLE) patients. EnHERV allowed the analysis of how different types of LTRs were differentially associated with specific gene expression profiles in particular disease conditions for further studies into their mechanisms and functions.

## Introduction

The human genome carries virus genetic content and is therefore part virus, similarly in various eukaryote genomes [1]. They have been known as interspersed repetitive sequences (IRSs) or transposable elements (TEs) because they can be copied or cut and then placed in other regions of the human genome. TEs are found in approximately 45% of the human genome. The ability of TEs to move within genomes impact host genome evolution [2]. Many recent studies have researched abilities of these element and how they contribute in their host gene regulation activities [3]. TEs can be classified as DNA transposons or retroelements and they encompass about 2.8% and 42.2% of the human genome, respectively [4]. Retroelements can be divided into two groups based on the presence or absence of long terminal repeats (LTRs). There are two types of non-LTR retroelements, short interspersed nuclear elements (SINEs, e.g. ALU) and long interspersed nuclear elements (LINEs), which are present in high numbers. The majority of LTR retroelements are derived from human endogenous retroviruses (HERVs), and about 8% of the human genome is made of LTR retrotransposons. While a variety of LTR retrotransposons have been identified, only vertebrate-specific endogenous retroviruses (ERVs) are known to be active in mammalian genomes [5]. Among them, HERVs have similar genomic structures to proviruses but contain a large number of mutations that have accumulated over evolution, especially in internal genes [1,2]. Some remaining HERV elements in a host genome are still active in their host genome.

The abundance and distribution of HERVs have been well characterized [6,7]. They have been known as junk DNA for a long time but more and more studies support their important regulatory function in the human genome e.g., the long terminal repeats of HERVH function as enhancers and a nuclear long noncoding RNA required to maintain hESC identity [8] and their contribution into the core regulatory network of embryonic stem cells [9]. The HERV fragments that remain in the human genome still have the ability to produce functional retroviral proteins e.g., the HERVW GAG protein detected in human brain, HERV-W7q ENV (Syncitin) expressed in placenta, and HERV-K (HML-2) loci encode retrovirus-like proteins expressed in tumor [10–12]. Moreover, their LTRs were shown to function as parts of regulatory sequences e.g., HERV-K LTR found as coexpression with MITF-M in malignant melanomas [3], MER21A/ERV1 acted as a primary promoter of *HSD17B1* in ovary and placenta [6], expression of *ZNF80* zinc-finger gene was driven by a solitary LTR of ERV9 [11]. A genome-wide screen identified more than 20,000 candidate regulatory regions derived from retrotransposons in the human genome and more than 2,000 examples of bidirectional transcription, emphasizing the regulatory role of retrotransposon in the mammalian genome [13]. Bioinformatic profiling and high-throughput experiments help speed up the discovery and one recent study identified ~110,000 regulatory active HERV elements that might impact the molecular function in human cells [14].

Generally, a complete HERV element is composed of two LTRs flanking a set of internal retroviral genes and can be represented as LTR1-Internal-LTR2. HERVs are frequently incorrectly annotated as complete elements because of the massive accumulation of insertions and deletions in HERV sequences. As a result, HERV annotations are often displayed as a number of fragments of an element rather than as a unified sequence with gaps [15]. Solitary LTR is the most abundant HERV annotation in the human genome because the recombination event between the 5′ and 3′ LTRs of a full-length provirus results in the loss of the internal sequence. The structure and distribution of TEs relative to other genes in the genome may help detect genomic elements that contribute to the development of phenotypic differences between disease and healthy individuals. The role of HERVs in human disease has been discussed especially in cancer and numerous autoimmune, neurological and infectious diseases. The expression level of HERV-E gag (group antigen) was found to be increased in peripheral blood mononuclear cells (PBMCs) of systemic lupus erythematosus (SLE) patients and increased HERV-K gag gene expression was reported in rheumatoid arthritis patients [16]. Furthermore, HERV-E gag transcription correlated with blood plasma concentrations of anti-U1 ribonucleoprotein (RNP) and anti-Sm antibodies in SLE patients. A HERV element was shown to participate in splicing of pre-mRNA to mRNA in SLE patients [17]. Nakkuntod and colleagues [18] examined the methylation status of two HERV-E and HERV-K sequences in lymphocytes from patients with SLE and found that hypomethylation of specific HERVs was a feature for SLE patients. One hypothesis is that lower methylation levels allow for expression of HERV genes, which may have some biological consequences. For example, 1) aberrant HERV transcripts and their protein products might lead to the production of autoantibodies due to molecular mimicry, 2) HERV mRNAs might serve as foreign nucleic acids and stimulate an abnormal immune response via endogenous immune receptors, or 3) regulatory regions, such as LTRs, in the HERVs can affect neighboring gene expression. Investigating the possible relations between a gene set and HERVs is important in identifying novel disease pathogenesis.

Although HERVs have been known for more than two decades, there is only a limited number of databases that facilitate finding HERVs in the human genome. Existing databases including 1) HERVd [19], designed to search for the location of HERV elements in the genome but information in the tool are quite outdated. RepeatMasker in HERVd is based on the 09/20/2000 version. 2.) Transpogene [20], which allows users to search for intragenic transposable elements in human transcripts. This version of human genome is based on NCBI build 36.1 (UCSC hg18). None provide an enrichment analysis function for gene of interest. Moreover, the location of HERVs in the human genome relative to coding exons can affect their function as well; therefore, we designed EnHERV to provide HERV neighboring gene information as intergenic and intragenic HERV elements. Not only does it provide HERV location, it also makes available the orientation of HERV elements and types of truncation patterns which result from the defragmentation process. Furthermore, EnHERV allows the user to define a distance of intergenic HERV elements from their neighboring genes from ranges of 1 to 100 kbs. Besides the search function in EnHERV, the enrichment analysis function provides association analysis between designed HERV characteristics and their neighboring genes in certain gene expression conditions.

## Results and discussion

### HERV identification

**Fig 1:**
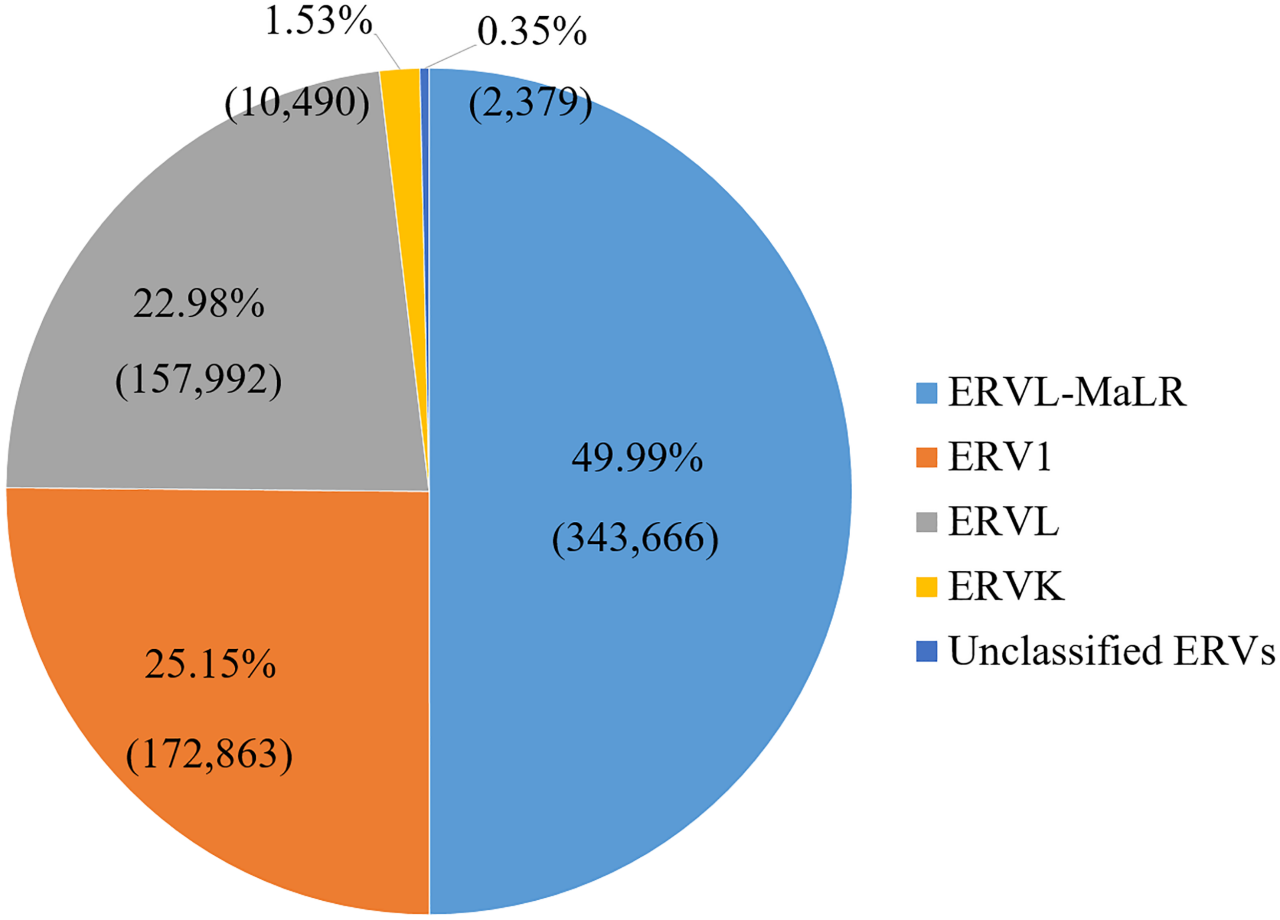
HERV superfamily distribution. Percentage and number of copy of each HERV superfamily in hg19/GRCh37 genome. 687,420 HERV elements were distributed into 5 groups including 4 superfamilies and one unclassified group. Almost 50% of HERV elements in the human genome belongs to the ERVL-MaLR superfamily.

Only data of the main chromosomes (chromosome 1–22, X, and Y) were included in the analysis because comprehensive genome annotation information is only available for the main chromosomes. We retrieved 687,420 HERV elements from a total of 5,298,130 repeat sequence records in the human hg19/GRCh37 genome (12.97% of the repeat sequences). Most of UCSC cross-reference sequences in the 24 main chromosomes (94.91% of total UCSC known genes) contained HERV elements. The cross-reference sequence annotation (kgXref table) was used to convert UCSC known genes to HGNC official gene names. We investigated the association of HERV under various disease conditions in this study. A list of HERV superfamilies and families is shown in Table 1. The association was done on three levels. For superfamilies, all four superfamilies were investigated. While some HERV families and individual HERVs were selected to represent their members in the group as listed in Table 1. The proportion of HERV fragments in the annotation data is shown in Fig 1. ERVL-MaLR is the most abundant HERV in the human genome. While only a little ERVK are present in our genome.

**Table 1:** Solo LTRs used in the enrichment analysis in EnHERV.

Superfamily	Family	Name/Group
ERV1	ERV9	LTR12, LTR12C
	HERVH	LTR7
	HERVW	LTR2, LTR2B, LTR2C
	HUERSP1	LTR8
	LOR1	LOR1a
	MER39	MER39
	MER4	MER4C
	MER52	MER52A
	MER57	MER57B1
ERVK	HERVK10/HERVK (HML-2)	LTR5_Hs
	HERVK11/HERVK (HML-8)	MER11C
ERVL	HERV16	LTR16A1
	HERVL33	LTR33
	HERVL52	LTR52
	HERVL66	LTR66
ERVL-MaLR	MLT	MLT1D, MLT2B3
	MST	MSTD
	THE1	THE1A, THE1B, THE1C, THE1D

In a post-processing step, we used REannotate [21] to defragment the HERV annotations before using them in EnHERV. Defragmentation was based on distance and orientation between fragments and HERV families. Components of HERV elements were separated into three parts of the complete genomic structure, which can be symbolized as LTR1-Internal-LTR2, where LTR1, Internal, and LTR2 represent an upstream LTR, an internal sequence, and a downstream LTR, respectively. Intactness ratios of the elements, which indicate how complete an element is, were also provided by REannotate. Typically, this value was calculated from the fraction of the reference sequence that matched in the query sequence.

**Fig 2:**
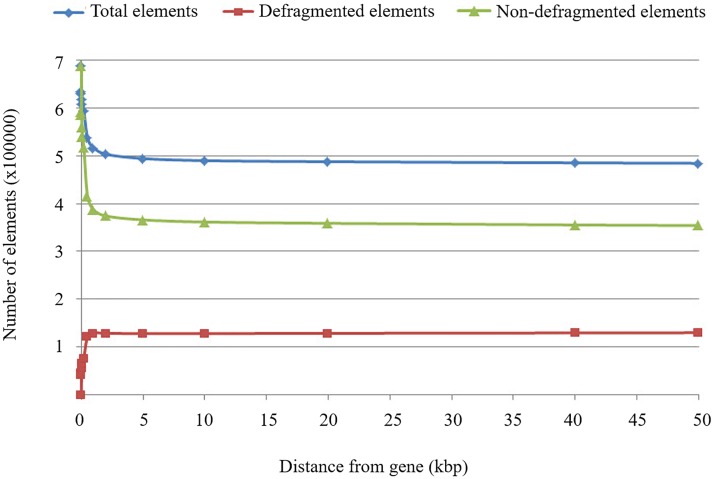
Number of defragmented and non-defragmented elements. Graph shows a number of defragmented and non-defragmented elements in relation to distance parameters.

The maximum distance is an important REannotate parameter, which is used to set the greatest distance allowed between two HERV fragments for them to be joined into the same element. REannotate was run several times using different distance parameters to determine the sensitivity of this value as mentioned in materials and methods. Defragmented elements are HERV elements that originated from combining more than one fragment together; the non-defragmented elements were composed of only one fragment; and the total number of all elements that resulted from defragmentation was the sum of the numbers of the defragmented and non-defragmented elements. Rate changes of all elements resulting from defragmentation using different distance parameter values are illustrated in Fig 2. Numbers of all elements and non-defragmented elements tended to decrease when the distance parameter was increased because more single fragments had to be used in the joining events, which resulted in more defragmented elements.

The number of all elements tended to change rapidly at smaller distances. This may suggest that the optimal distance should not be too small because many defragmented elements would be ignored. We also measured the rates of change of the number of all elements by varying distance parameters to determine the distance at which there were no changes in the number of all elements. Although, no standard value was available for the distance parameter in defragmentation, suggestions from previous studies have varied between 500 bps to 30 kb [22,23]. Based on these results in Table 2, we set the appropriate value of the distance parameter as 500 bps to cover 122,437 defragmented elements (94.14% of the maximum number of defragmented elements). In total, we obtained 537,061 HERV elements from the defragmentation process, which were 10.26% of repeat sequences in UCSC hg19 main chromosomes. Of the total number of HERVs, 86% were annotated as LTRs and the remaining 14% were annotated as internal genes.

**Table 2:** The number of defragmented elements, resulting from HERV defragmentation using REannotate with different values of distance parameters, and their coverage percentages.

The maximum distances (bp)	The number of defragmented elements	Coverage percentages
10	42,489	32.67%
20	45,678	35.12%
50	57,284	44.04%
100	66,500	51.13%
200	77,070	59.26%
300	91,343	70.23%
400	111,250	85.53%
500	122,437	94.14%
1,000	129,607	99.65%
2,000	129,007	99.19%

A HERV family and group manipulation process was performed to avoid redundant family names in repeat annotations. According to the four superfamilies, we obtained a total of 413 groups of HERVs, which were classified into 133 HERV families. The full annotation is listed in S4 Table. Notably, most of the existing HERVs were rarely found as complete elements due to the accumulations of insertions and deletions in their sequences over time.

**Fig 3:**
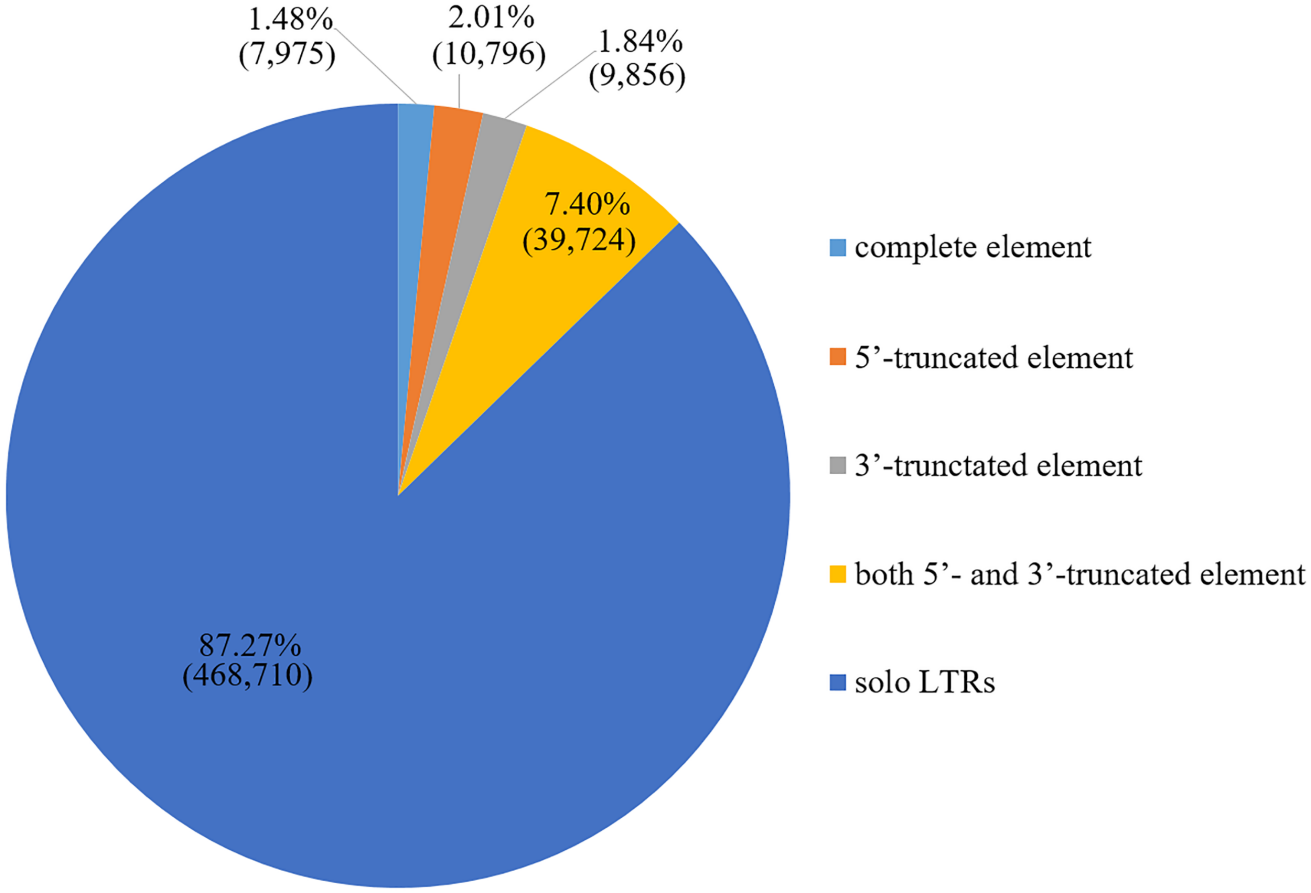
HERV truncation pattern distribution. Percentage and number of each HERV truncation pattern in the HERV elements resulting from the defragmentation process.

According to the 5′-LTR1-Internal-LTR2-3′ structure of the HERVs, five classification types of the truncation patterns were detected: 1) complete, 2) 5′-truncated, 3) 3′-truncated, 4) both 5′- and 3′-truncated elements, and 5) solitary or solo LTRs. The proportion of each truncation type is shown in Fig 3. The majority of truncation patterns were solo LTRs. Since LTRs may drive the transcription of adjacent host genomic sequences [24], we developed EnHERV to analyze various HERVs patterns that may be associated with gene expression patterns in certain disease conditions.

The manipulated HERV defragmented elements from the REannotate output and the selected gene annotations were mapped together. HERV defragmented elements located between 100,000 bps upstream from the transcription start site and 100,000 bps downstream from the transcription termination site of the neighboring gene were considered as HERV neighboring genes as they have been known to act upon genes up to 70–100 kb away [25–27]. The results of this integration of HERV elements and human genes showed that 382,662 HERV elements (71.24% of the total 537,061 elements) were identified in 73,645 gene isoforms (99.98% of the total 73,660 UCSC gene isoforms), which may imply that most gene isoforms in the human genome contain HERV elements near or within the genes.

### HERV neighboring gene profiles

We identified 382,662 HERV elements (71.24%) as neighboring loci of 73,645 gene isoforms (99.98%) in the human genome. There was a slight bias in the anti-sense orientation of HERV elements and their neighboring genes comparing to sense orientation which is 206,637 elements (54.05%) and 176,025 elements (45.95%), respectively. 5′ upstream and 3′ downstream regions of the gene which are 160,718 elements (42%) and 168,371 elements (44%), respectively. Most of the remaining intragenic HERVs were located in introns. Solo LTRs were the most abundant HERV structure.

### The EnHERV database

Users can access EnHERV at http://sysbio.chula.ac.th/enherv. EnHERV provides two search functions: 1) Search by gene(s) and 2) Search by HERV characteristics, including HERV superfamily, family, group/name, location in genome, distance from gene (which user defines distance as an option), orientation, and structure completeness. Search results are displayed in table format. EnHERV provides a link to the UCSC genome browser for visualizing the region of the genome structure for the search results. The EnHERV database also allows users to download results for downstream analysis. In the search by gene name option, EnHERV will try to auto-complete a user-input gene name that contains or is surrounded by HERVs. Moreover, EnHERV allows users to download all the records from the database for further customized analyses.

**Fig 4:**
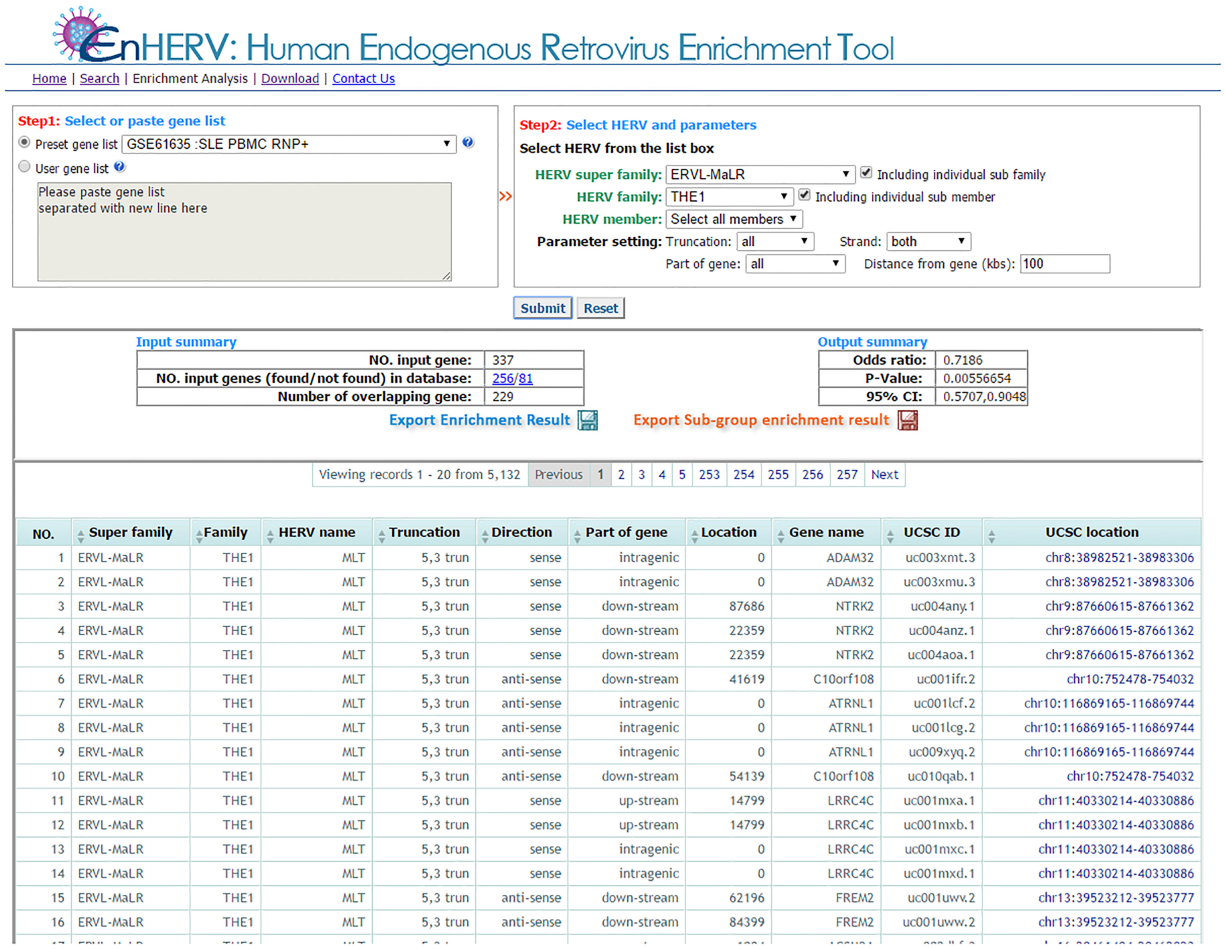
The enrichment analysis page. EnHERV’s enrichment analysis page. EnHERV gives the Fisher’s exact, P-value and odd ratio of the designed enrichment analysis. EnHERV also provides parallel analysis for sub-selected HERV superfamily/family.

In addition to a searching function, EnHERV also provides an enrichment analysis function that allows users to perform an enrichment analysis between genes with user-specified HERV characteristics and a user-defined gene list. EnHERV will calculate Fisher’s p-values and odds ratios for analysis results as mentioned in the methods section. Genes containing the selected HERV characteristics will be displayed in a result table, which users can download for further investigation. Furthermore, EnHERV also allows users to perform enrichment analyses for all the members of the selected HERV superfamily/family at once. Users can then save the enrichment analysis output as illustrated in Fig 4.

### Analysis of solo LTRs in cancer and autoimmune diseases

Hypomethylated HERVs have been found to be active in cancer and autoimmune diseases [18,28]. Distinct isoforms or gene silencing due to global hypomethylation of HERVs has been reported in various diseases, particularly in cancers [29,30] and SLE [31]. Because HERVs can control neighboring genes by either up- or down-regulating their expression, we developed a model to identify genes that were associated with HERVs in the genome and were differentially expressed in various diseases. This information can serve as a screening tool for further studies of candidate genes that might be regulated by HERVs.

As an example, we studied differentially expressed gene patterns in various specific tissue and disease conditions. Association analyses were performed in 49 disease conditions as listed in S1 Table. Significant associations were analyzed between various gene expression conditions and the four HERV superfamilies (S2 Table) and 25 individual HERVs that represent each family (S3 Table).

**Fig 5:**
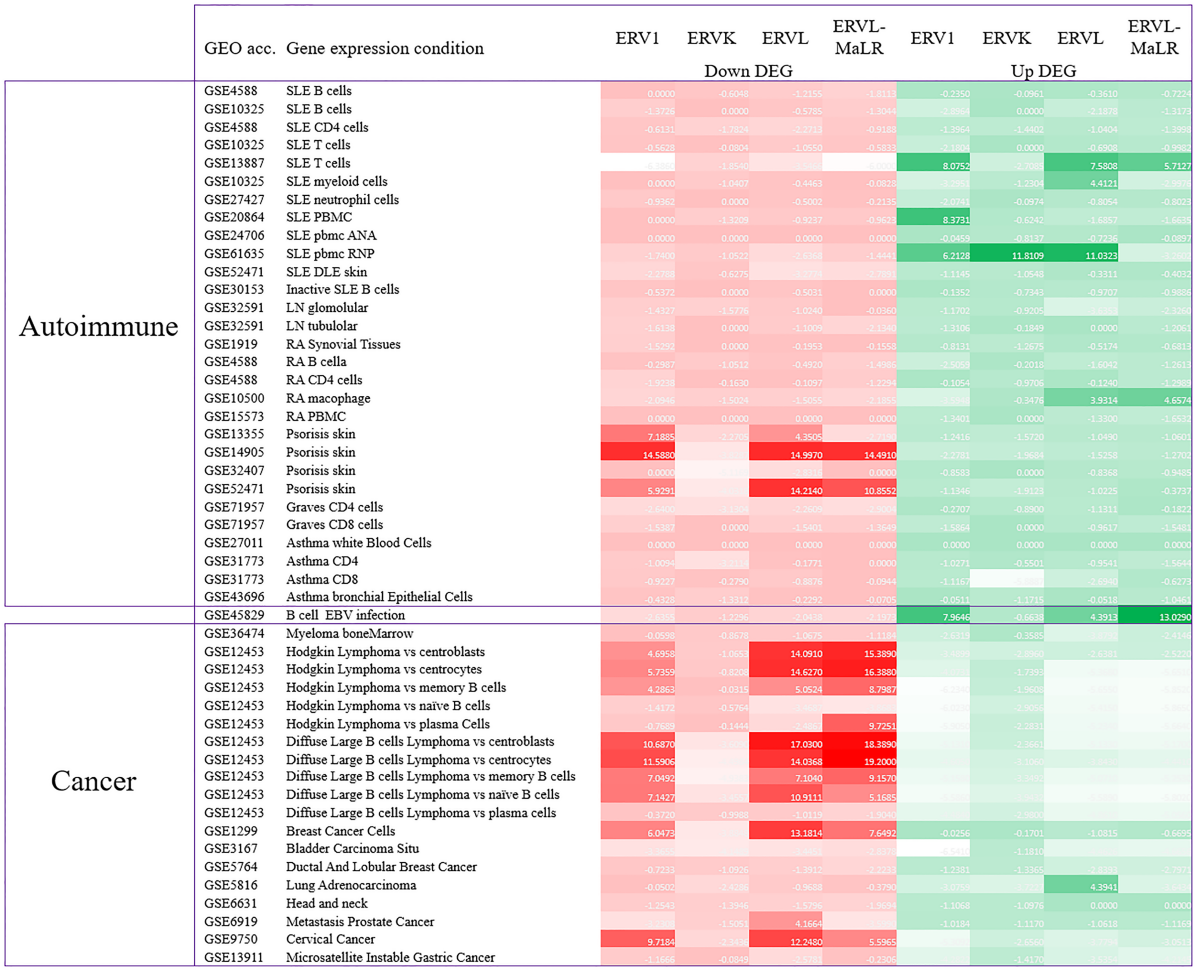
Association heatmap between intragenic HERV and disease conditions. A different intragenic HERV association pattern between cancer and auto-immune disease was identified. Intragenic HERVs were strongly associated with down-regulated genes in cancer. In contrast they are highly associated with up-regulated genes in immune cells under auto immune disease conditions.

First, significant associations were detected mostly with the intragenic HERVs (S2 and S3 Tables). Second, significant associations with most gene expression conditions were found (Fig 5 and S2 Table). Heatmap in Fig 5 represents the association level of HERVs. The minus of log P-value were calculated to represent the association level of HERVs and certain gene condition. The darker color represents a stronger association level. Red represents an association to genes in down-regulation conditions while green represents genes in up-regulation conditions. Furthermore, with the P-value < 0.001 and odds ratio > 1 cutoff criteria was mainly with the ERV1, ERVL, and ERVL-MaLR superfamilies but not with the ERVK superfamily (S1 Fig). Third, the pattern of association was different between various disease conditions. We found that intragenic HERVs were associated with down-regulated genes in most cancer conditions and in psoriatic skin tissue and associated with up-regulated genes in immune cells from SLE patients, macrophages from RA patients, and Epstein-Barr virus (EBV) infected B cells (Fig 5, S1 Fig and S2 Table).

This finding is interesting because LINE-1s, which are another type of IRS, have also been shown to be associated with down-regulated genes in cancer. It was suggested that LINE-1s, which were found to be globally hypomethylated in cancer tissue, might control neighboring genes by acting as antisense RNAs [32]. Interestingly, our analysis with HERV in this study, another IRS element that was also reported to be hypomethylated in cancer tissues, revealed that they had the same antisense direction. Further proof is required to determine if HERVs can control neighboring genes in the same way as LINE-1s. Another observation is the similar pattern of association in cancer and psoriatic skin tissues. We previously reported LINE-1 hypomethylation in keratinocytes from psoriatic patients and observed that genes with LINE-1 in their vicinity were down-regulated more than genes without LINE-1, similar to what was observed in cancer [33]. We found a similar association with HERVs in the present study. These findings are interesting because keratinocyte proliferation, a characteristic similar to cancer cells, is the main feature in psoriatic skin tissue. Mechanisms of LINE-1- and HERV-mediated gene regulation in cancer cells and keratinocytes remain to be studied further.

Similar to reports in cancer tissues, we have reported global hypomethylation of IRS including LINE-1 and certain HERV types in immune cells of SLE patients [18,34,35]. We hypothesized that these IRS elements could also play roles in controlling neighboring genes. We have reported previously that the up-regulated genes in neutrophils from SLE patients were significantly associated with genes containing LINE-1 [34]. When analyzing genes containing HERVs in the current study, we observed the same association with up-regulated genes in immune cells from SLE. It should be noted that significant associations were observed with only 3 out of 11 microarray data sets, which might be due to disease heterogeneity of the diseases and more data should be further analyzed to validate this observation. However, this observation might suggest a difference in the pathogenesis of how LINE-1 and HERVs affect gene regulation in cancer and SLE. A likely hypothesis is that aberrant LINE-1 or HERV regulation in SLE leads to up-regulation of these retroelement-like transcripts, thereby stimulating immune receptors in the cells and results in the activation of immune genes, including the well-known interferon responsive genes [36–38]. The role of HERV in regulating a gene in SLE was demonstrated by the finding that an alternative transcript of CD5 was regulated by a neighboring HERV-E in SLE B cells [31]. It is possible that these LTRs could function as promoters, enhancers, or cause alternative splicing. Furthermore, TEs also occur in more than two thirds of mature long noncoding RNAs (lncRNA) transcripts and account for a substantial portion of total lncRNA sequence (~30% in human). lncRNAs may be used for various tasks, including post-transcriptional regulation, organization of protein complexes, cell-cell signaling and allosteric regulation of proteins [39–42]. The exonic TEs were proposed to act as RNA domains that are essential for lncRNA function called Repeat Insertion Domains of LncRNAs (RIDLs) [43]. Our analysis also showed that HERVs were associated with up-regulated genes in B cells with EBV infection similar to the HERV association in SLE. This observation is interesting because the EBV has been implicated as a major risk factor for SLE. Interestingly, we found no significant associations between HERVs and immune cells from other immune-mediated diseases that we analyzed, including asthma, Graves’ disease, and rheumatoid arthritis (except for macrophages). Our results indicate that the mechanism in SLE that involved HERVs works mainly in the immune cells and have some specificity with certain HERVs.

The results of individual LTR analysis showed that not all the LTRs from the same superfamily showed significant associations. Our review regarding the role of specific LTR that control certain genes is summarized in S5 Table. Using EnHERV, we would like to give some examples in SLE as the following. The associations were detected between intragenic ERV1, ERVL, and ERVL-MaLR superfamilies with up-regulated genes in SLE T cells and in PBMCs with RNP+ conditions; however, no such associations were found for the ERVK superfamily (S2 Table). This finding correlated with the results of our previous study that hypomethylation of HERV-E, but not HERV-K, was detected in SLE CD4+ T cells [18]. This specific hypomethylation was also associated with up-regulation of HERV-E transcripts in CD4+ T cells [44]. Moreover, our results showed a particularly strong association of SLE with RNP+. This is consistent with the reported sequence homology between HRES-1 and the 70-kDa *gag*-related region of sn-RNP, which supports the suggestion that a possible mechanism in etiopathogenesis of SLE is the induction of a cross-reaction between the two proteins by autoantibodies. EnHERV could help screen for the specific LTR pattern and type that is involved in a disease of interest so that the mechanisms and functions can be further studied.

## Materials and methods

To build EnHERV we used genome data from the UCSC Table Browser [45] as the core information.

The UCSC genome annotation database for the February 2009 assembly of the human genome (hg19, GRCh37 Genome Reference Consortium Human Reference 37 (GCA_000001405.1)) was used in the analysis. Three UCSC tables were used in our analysis. 1) The human repeat annotations, RepeatMasker version open-3.2.7 (rmsk table listed in RepeatMasker track/ Repeats group) containing 5,298,130 repeat records (last updated: 2009-04-24). HERV names in EnHERV were based mainly from the rmsk table. 2) The human gene annotations (knownGene table), and 3) The cross-reference IDs (kgXref table), were used for mapping UCSC gene to gene symbol. The knowGene and kgXref tables contain 82,960 UCSC IDs (last updated: 2013-06-14). Both tables were listed in UCSC Genes track/Genes and Gene Predictions group. Gene information included sequences of the 24 main chromosomes (chromosome 1–22, X, and Y), 59 unplaced contigs, and nine haplotype chromosomes.

Five HERV classes were defined based on the Repbase classification system [46], namely i) class 1 superfamily (ERV1), which contains the HERVs related to gamma retroviruses such as murine leukemia virus (MLV) and baboon endogenous virus (BaEV); ii) class 2 superfamily (ERVK), which contains beta retroviruses including mouse mammary tumor virus (MMTV); iii) class 3 superfamily (ERVL), which is distantly related to spuma retroviruses; iv) the mammalian apparent LTR-retrotransposons (ERVL-MaLR), which is considered as an additional class [47,48]; and v) a group of unclassified fragments, which contain other HERV-like sequences. The full list of these HERVs is shown in S4 Table.

The computational identification and annotation of the HERVs in the reference genome was generally incomplete and the HERV element is usually annotated as a separated fragment. Therefore, the HERV defragmentation process was done by REannotate for joining HERV fragments which belonged to the same HERV element into a single element. The REannotate defragmentation program is based on distance, orientation between fragments, and membership of the same HERV family. The distances in base pairs that we tested were 10, 20, 50, 100, 200, 300, 400, 500, 1 k, 2 k, 5 k, 10 k, 20 k, 40 k, and 50 k. The numbers of defragmented elements, non-defragmented elements, and total elements were determined for each of the distance parameters tested. The equivalent REannotate name list and other information were retrieved from Repbase Update [46]. To manipulate the redundant family names in the REannotate output, the information of each family name was extracted from the comment lines described in the Repbase. Moreover, the information of previous annotated name of LTRs and their internal sequence was also available in Repbase. It was used as reference information in the manipulation process. After defragmentation, the defragmented HERV elements were mapped to every annotated gene isoform by their location in the human genome.

EnHERV was constructed as a web database tool for easy access by users. EnHERV uses PHP to generate dynamic HTML, CSS and Javascript. A MySQL was implemented for recording HERV neighboring gene information. The enrichment function was implement by Python programming language. Two major functions were implemented in EnHERV which are search function and enrichment analysis function. The search function allows users to connect to the pre-built HERV profile database, as described above. Two search options are provided: search by gene(s) and search by HERV characteristics. Seven HERV characteristics can be input: 1) HERV superfamily, 2) HERV family, 3) HERV name, 4) HERV orientation, 5) HERV distance from their neighboring gene, 6) HERV location in gene, and 7) HERV completeness type. The enrichment analysis function implements Fisher’s exact test to test the nonrandom associations between user-defined gene or preset gene lists and genes that contain specific HERV characteristics. Fisher’s exact p-value was calculated to examine the significance of the association from contingency table between genes in the giving list with and without specific HERV characteristics in defined conditions and human UCSC genes with and without the same specific HERV characteristics.

HERVs have mainly been reported to be involved in cancer and autoimmune disease, ten different cancer and autoimmune experiments were retrieved from the gene expression omnibus (GEO) [49,50] and built in to EnHERV as sample gene lists. We hypothesized that HERVs could affect the expression of their neighboring genes by either up- or down-regulation, and that the association may be in a specific direction and location. We performed an enrichment analysis in EnHERV to detect the associations between specific HERV properties and various disease conditions. We retrieved 49 GEO accessions from the NCBI GEO database (https://www.ncbi.nlm.nih.gov/geo/) and classified the gene expressions into 49 conditions, including autoimmune and other disease conditions, as shown in S1 Table. Differentially expressed genes were identified using the GEO2R function (http://www.ncbi.nlm.nih.gov/geo/geo2r/). Genes were considered as differentially expressed for P-values with Benjamini & Hochberg adjustment ≤ 0.05 and fold-changes >1-fold.

We tested the association between various disease conditions and four HERV superfamilies. We also used 25 individual HERVs that represent each family for the enrichment analysis (Table 1). Most of the HERVs were found to be expressed HERV elements in previous reports [51–56]. HERVs and disease conditions were considered as associated events when Fisher’s exact p-value was <0.001 and the odds ratio was >1.

## Conclusions

Many reports have supported the idea that epigenetics plays an import role in disease pathogenesis [57–61]. Previous publications have shown that TEs can alter the expression of their nearby genes as a result of methylation imbalances. Therefore, we investigated the association between HERVs and gene expression under various disease conditions, especially in SLE. First, we developed EnHERV as a HERV database and enrichment tool using repeat and human genome information from Repbase and the UCSC Table Browser. EnHERV is available at http://sysbio.chula.ac.th/enherv/. EnHERV provides searches by gene names or HERV characteristics and also allows users to perform enrichment analysis between gene lists of user interest and specific selected HERV characteristics. Thousands of enrichment analyses were performed in this study. The results suggested that certain disease conditions were associated with specific LTR types. The EnHERV database and built-in functions will help in further understanding the pathogenesis of not only SLE, but also other diseases where HERVs might be involved in their pathogenesis.

## Supporting information

S1 Fig The highly HERVs diseases signification association. With the P-value < 0.001 and odd ratio > 1 cutoff criteria, the ERV1, ERVL, and ERVL-MaLR superfamilies but not with the ERVK superfamily show the different pattern various disease conditions.
(TIF)

S1 File Data availability statement. (DOCX)

S1 Table Data retrieved from the NCBI GEO database (https://www.ncbi.nlm.nih.gov/geo/) and disease conditions into which the differentially expressed genes were used in demonstration study. (DOCX)

S2 Table Association analysis results between various gene expression conditions and the four HERV superfamilies. Significant data with odds ratio >1 and Fisher’s p-value <0.001 are indicated in green letters for up-regulated genes and red letters for down-regulated genes.
(XLSX)

S3 Table Association analysis results between various gene expression conditions and individual HERVs that represent each superfamily. Significant data with odds ratio >1 and Fisher’s p-value <0.001 are indicated in in green letters for up-regulated genes and red letters for down-regulated genes.
(XLSX)

S4 Table Full list of HERVs in the EnHERV database. (DOCX)

S5 Table Evidences of LTR involved in gene expression. (DOCX)
