# Supplementary figures and images for: EnHERV: Enrichment analysis of specific human endogenous retrovirus patterns and their neighboring genes
Source: PLoS One. 2017 May 4;12(5):e0177119. doi: 10.1371/journal.pone.0177119 (PMC5417679; doi:10.1371/journal.pone.0177119)

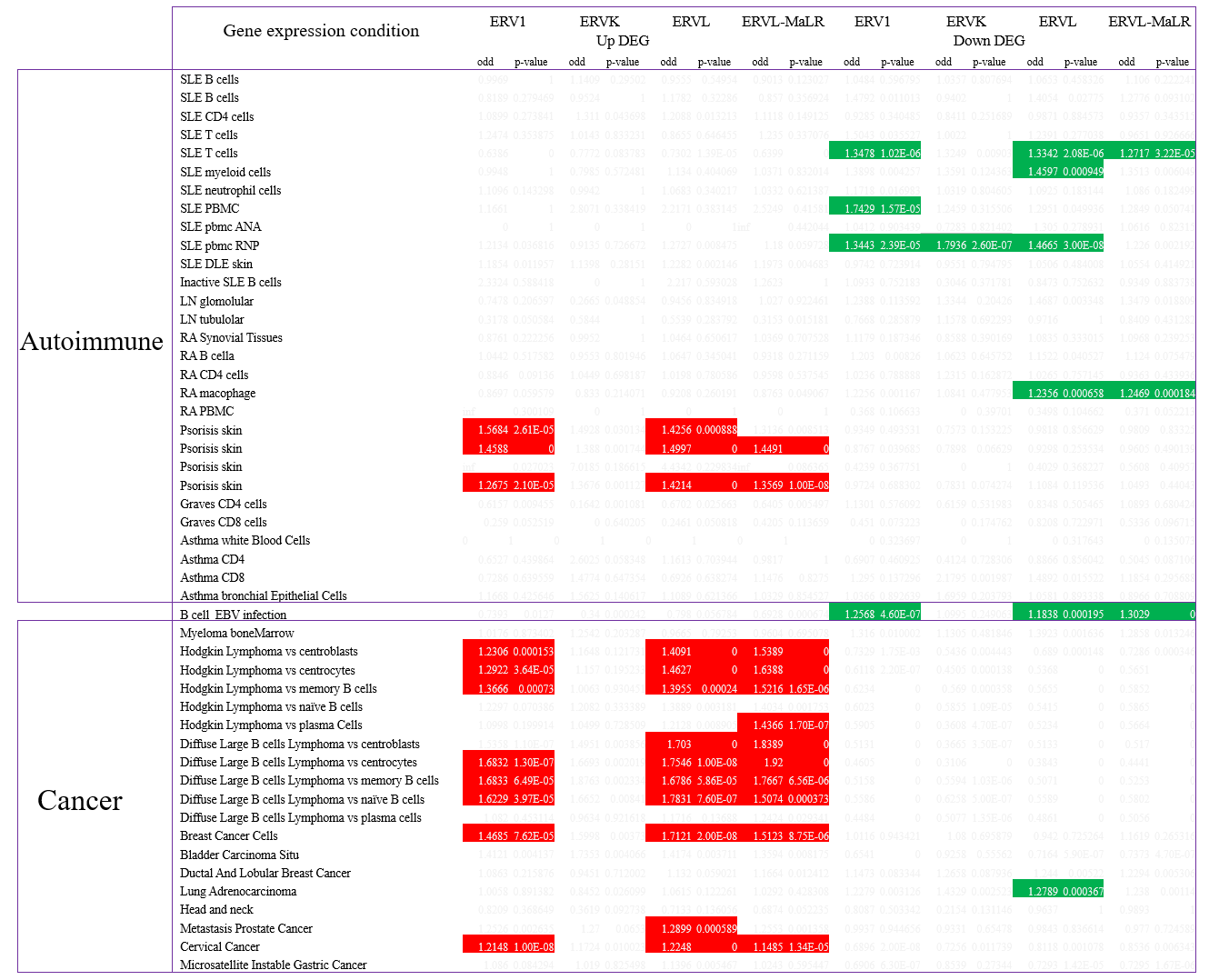

Supplement: S1 Fig — With the P-value < 0.001 and odd ratio > 1 cutoff criteria, the ERV1, ERVL, and ERVL-MaLR superfamilies but not with the ERVK superfamily show the different pattern various disease conditions. (TIF) [file pone.0177119.s001.tif]
