# Supplementary material for: EnHERV: Enrichment analysis of specific human endogenous retrovirus patterns and their neighboring genes
Source: PLoS One. 2017 May 4;12(5):e0177119. doi: 10.1371/journal.pone.0177119 (PMC5417679; doi:10.1371/journal.pone.0177119)
Supplement: S1 File — (DOCX) [file pone.0177119.s002.docx]

**Data Availability Statement**

To study the association analysis between specific HERV characteristic and their neighboring gene as shown in this publication, two components below are required;

- HERV neighboring gene according to specific characteristic. HERV LTRs that used in this paper were listed in **Table 2** in the publication. Four solo LTR characteristics were tested;
  - Sense solo LTR
  - Anti-sense solo LTR
  - Intragenic solo LTR
  - Intergenic solo LTR
- Up- and down-regulated genes in various disease condition (List in S1 Table) were retrieved from GEO. They were also provided as example gene lists in the download section. The download link is http://sysbio.chula.ac.th/enherv/database/example_gene_lists.zip

To perform enrichment analysis, user must perform each individual pair. For example, if we are interested in down regulated gene under SLE PBMC RNP+ condition, we have to get the gene list from related file.

- Down regulated genes were listed in folder “down_regulated” in the file “down_SLE_GSE61635_pbmcRNP.txt”

Go to **Enrichment Analysis** tab in EnHERV

Step 1. Put genes of interest in the **User gene list** box

Step 2. Select desired HERV characteristic. For example

To analyze **ERV1 super family** with specific characteristic as specified below.

- Truncation : Solo LTR (which is the characteristic that was analyzed in this publication)
- Strand : Both
- Part of gene : Intragenic (Note that the distance from gene option is only available for intergenic characteristic)

The input page will be shown as Figure below.


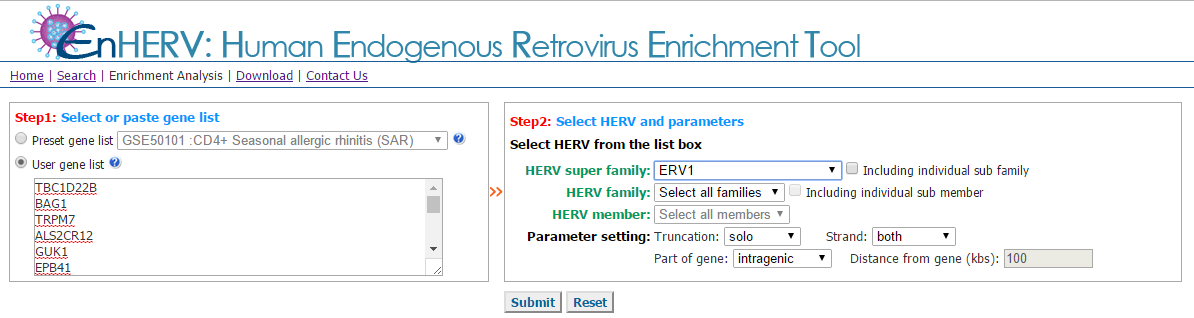


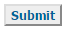
Once user click , The output will be shown as table below.


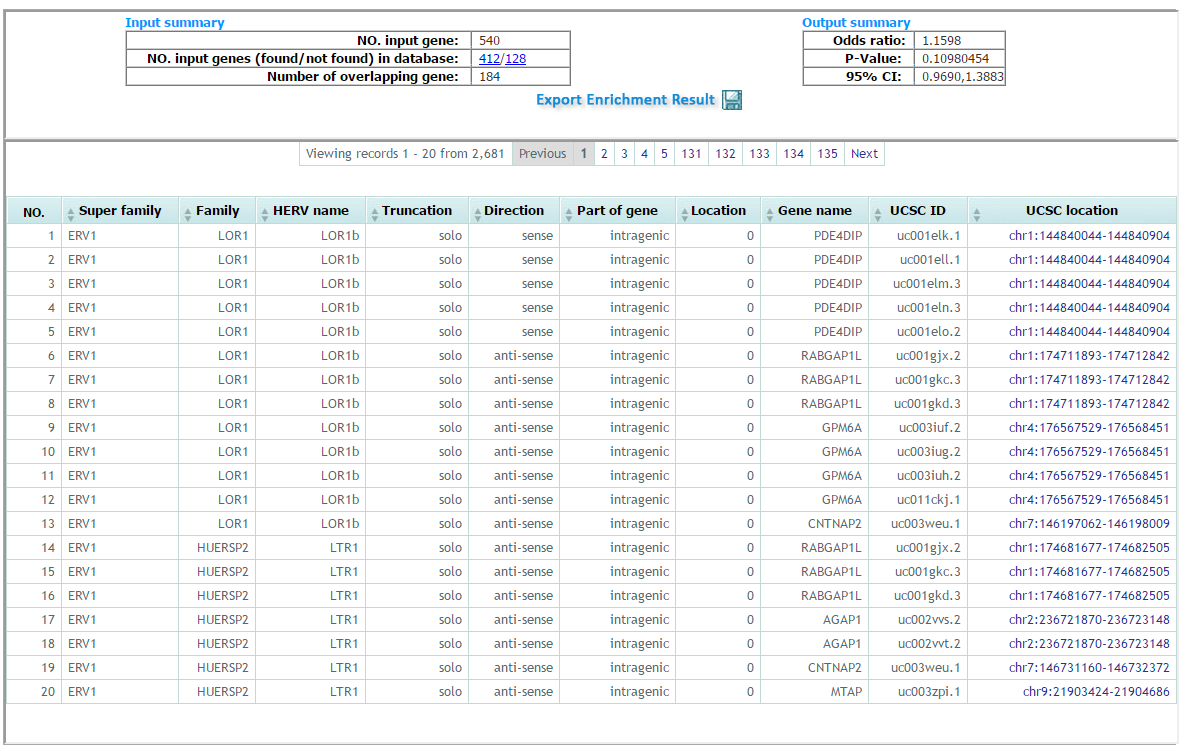


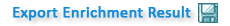


User can save the enrichment analysis result by click


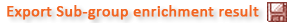
If “**Including individual sub family or member”** option was selected, all enrichment analysis will be calculated for all sub families or members under the selected HERV. The sub-group enrichment result will appear. User can save the sub-group enrichment analysis result by click

The **odd ratio, p-value, and CI** for the association of down regulated genes from GEO accession SLE_GSE61635_pbmcRNP to ERV1 solo-LTR will be obtained from the program. We have performed analysis the same way with other characteristics of ERV1. The summary of the enrichment analysis of **all characteristics** in **ERV1** superfamily of **down regulated gene under SLE PBMC RNP+** condition were shown in table below.

| Superfamily | Characteristic | | | Enrichment result | | |
| --- | --- | --- | --- | --- | --- | --- |
|  | Truncation | Strand | Part of gene | odd | p-value | CI |
| ERV1 | Solo | Both | All | 0.1805 | 0 | 0.1480,0.2200 |
| ERV1 | Solo | Sense | All | 0.3277 | 0 | 0.2732,0.3931 |
| ERV1 | Solo | Anti-sense | All | 0.3668 | 0 | 0.3037,0.4432 |
| ERV1 | Solo | Both | Intragenic | 1.2134 | 0.0368163 | 1.0136,1.4525 |
| ERV1 | Solo | Both | Intergenic | 0.188 | 0 | 0.1543,0.2289 |

The enrichment analysis of **all characteristics** in **ERV1** superfamily of **up regulated gene under SLE PBMC RNP+** condition can also be analyzed the same way. All the results were summarized in table below.

| Superfamily | Characteristic | | | Enrichment result | | |
| --- | --- | --- | --- | --- | --- | --- |
|  | Truncation | Strand | Part of gene | odd | p-value | CI |
| ERV1 | Solo | Both | All | 0.116 | 0 | 0.1004,0.1341 |
| ERV1 | Solo | Sense | All | 0.2681 | 0 | 0.2340,0.3071 |
| ERV1 | Solo | Anti-sense | All | 0.2506 | 0 | 0.2186,0.2874 |
| ERV1 | Solo | Both | Intragenic | 1.3443 | 2.39E-05 | 1.1746,1.5385 |
| ERV1 | Solo | Both | Intergenic | 0.1221 | 0 | 0.1057,0.1410 |

The enrichment analysis of **all characteristic** in **ERV1** superfamily of **down** and **up regulated gene under SLE PBMC RNP+** condition was present as part of **S2 Table** in the publication (red box).


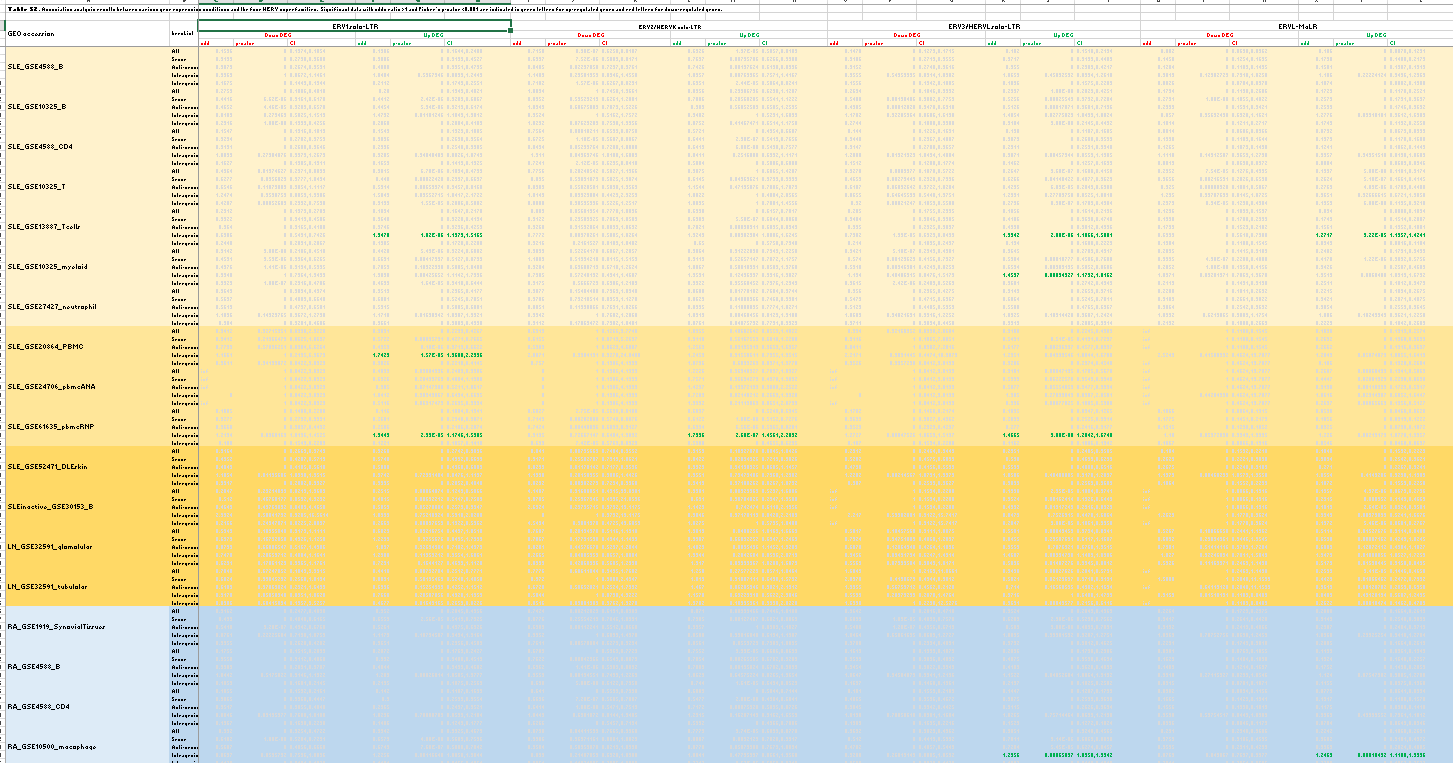


Note that the **S2 Table** shown in the publication was a summary of the enrichment analysis results between all 4 HERV superfamily and 49 up- and 49-down regulated gene conditions.

The association level between intragenic solo LTR characteristic and 49 up- and 49-down regulated gene condition (which is part of **S2 Table**) were shown as heatmap in **Figure 5** in EnHERV publication.

The enrichment analysis results of selected individual HERV member under 49 up- and 49 down-regulated gene conditions were performed the same way as above. All the analysis results were summarized in **S3 Table**.
