# Supplementary material for: EnHERV: Enrichment analysis of specific human endogenous retrovirus patterns and their neighboring genes
Source: PLoS One. 2017 May 4;12(5):e0177119. doi: 10.1371/journal.pone.0177119 (PMC5417679; doi:10.1371/journal.pone.0177119)
Supplement: S1 Table — (DOCX) [file pone.0177119.s003.docx]

**Table S1.** Data retrieved from the NCBI GEO database (https://www.ncbi.nlm.nih.gov/geo/) and disease conditions into which the differentially expressed genes were used in demonstration study.

| GEO accession | Disease condition |
| --- | --- |
| GSE4588 | SLE B cells |
| GSE10325 | SLE B cells |
| GSE4588 | SLE CD4 cells |
| GSE10325 | SLE T cells |
| GSE13887 | SLE T cells |
| GSE10325 | SLE myeloid cells |
| GSE27427 | SLE neutrophil cells |
| GSE20864 | SLE PBMC |
| GSE24706 | SLE pbmc ANA |
| GSE61635 | SLE pbmc RNP |
| GSE52471 | SLE DLE skin |
| GSE30153 | Inactive SLE B cells |
| GSE32591 | LN glomolular |
| GSE32591 | LN tubulolar |
| GSE1919 | RA Synovial Tissues |
| GSE4588 | RA B cella |
| GSE4588 | RA CD4 cells |
| GSE10500 | RA macophage |
| GSE15573 | RA PBMC |
| GSE13355 | Psorisis skin |
| GSE14905 | Psorisis skin |
| GSE32407 | Psorisis skin |
| GSE52471 | Psorisis skin |
| GSE71957 | Graves CD4 cells |
| GSE71957 | Graves CD8 cells |
| GSE27011 | Asthma white Blood Cells |
| GSE31773 | Asthma CD4 |
| GSE31773 | Asthma CD8 |
| GSE43696 | Asthma bronchial Epithelial Cells |
| GSE45829 | B cell EBV infection |
| GSE36474 | Myeloma boneMarrow |
| GSE12453 | Hodgkin Lymphoma vs centroblasts |
| GSE12453 | Hodgkin Lymphoma vs centrocytes |
| GSE12453 | Hodgkin Lymphoma vs memory B cells |
| GSE12453 | Hodgkin Lymphoma vs naïve B cells |
| GSE12453 | Hodgkin Lymphoma vs plasma Cells |
| GSE12453 | Diffuse Large B cells Lymphoma vs centroblasts |
| GSE12453 | Diffuse Large B cells Lymphoma vs centrocytes |
| GSE12453 | Diffuse Large B cells Lymphoma vs memory B cells |
| GSE12453 | Diffuse Large B cells Lymphoma vs naïve B cells |
| GSE12453 | Diffuse Large B cells Lymphoma vs plasma cells |
| GSE1299 | Breast Cancer Cells |
| GSE3167 | Bladder Carcinoma Situ |
| GSE5764 | Ductal And Lobular Breast Cancer |
| GSE5816 | Lung Adrenocarcinoma |
| GSE6631 | Head and neck |
| GSE6919 | Metastasis Prostate Cancer |
| GSE9750 | Cervical Cancer |
| GSE13911 | Microsatellite Instable Gastric Cancer |
