# Supplementary material for: EnHERV: Enrichment analysis of specific human endogenous retrovirus patterns and their neighboring genes
Source: PLoS One. 2017 May 4;12(5):e0177119. doi: 10.1371/journal.pone.0177119 (PMC5417679; doi:10.1371/journal.pone.0177119)
Supplement: S4 Table — (DOCX) [file pone.0177119.s006.docx]

**Table S4**. Full list of HERVs in the EnHERV database.

| **Superfamily** | **Family** | **Name/Group** |
| --- | --- | --- |
| **1. ERV1** | ERV9 | HERV9, LTR12, LTR12B, LTR12C, LTR12D, LTR12E, LTR12F |
|  | HERV1 | HERV1_I, HERV1_LTRa, HERV1_LTRb, HERV1_LTRc, HERV1_LTRd, HERV1_LTRe |
|  | HERV15 | HERV15, LTR15 |
|  | HERV17 | HERV17, LTR17 |
|  | HERV23 | LTR23, LTR44, LTR56 |
|  | HERV3 | HERV3, LTR4 |
|  | HERV30 | HERV30, LTR30 |
|  | HERV35 | HERV35I, LTR35, LTR35A, LTR35B |
|  | HERV38 | LTR38, LTR38B, LTR38C |
|  | HERV39 | LTR39 |
|  | HERV4 | HERV4_I |
|  | HERV43 | LTR43, LTR43B |
|  | HERV45 | LTR45, LTR45B, LTR45C |
|  | HERV46 | LTR46 |
|  | HERV49 | LTR49 |
|  | HERV70 | LTR70 |
|  | HERVE | HERVE, HERVE_a |
|  | HERVFc1 | HERVFc1, HERVFc1_LTR1, HERVFc1_LTR2, HERVFc1_LTR3 |
|  | HERVFc2 | HERVFc2 |
|  | HERVFH19 | HERVFH19 |
|  | HERVFH21 | HERVFH21, LTR21A, LTR21B |
|  | HERVH | HERVH, LTR7, LTR7A, LTR7B, LTR7C, LTR7Y |
|  | HERVH48 | HERVH48, MER48 |
|  | HERVI | HERVI, LTR10A, LTR10B, LTR10B1, LTR10C, LTR10D, LTR10E, LTR10G |
|  | HERVIP10 | HERVIP10F, HERVIP10FH, LTR10F |
|  | HERVP71A | HERVP71A, LTR71A, LTR71B |
|  | HERVS71 | HERVS71, LTR6A, LTR6B |
|  | Harlequin/HERVW | Harlequin_I, LTR2, LTR2B, LTR2C |
|  | HUERSP1 | HUERSP1, LTR8, LTR8A |
|  | HUERSP2 | HUERSP2, LTR1, LTR1B, LTR1C, LTR1D |
|  | HUERSP3 | HUERSP3, HUERSP3b, LTR9, LTR9B |
|  | LOR1 | LOR1, LOR1a, LOR1b, LTR26, LTR26B, LTR26E |
|  | LTR19 | LTR19, LTR19A, LTR19B, LTR19C |
|  | LTR24 | LTR24, LTR24B, LTR24C |
|  | LTR25 | LTR25 |
|  | LTR27 | LTR27, LTR27B |
|  | LTR28 | LTR28 |
|  | LTR29 | LTR29 |
|  | LTR31 | LTR31 |
|  | LTR34 | LTR34 |
|  | LTR36 | LTR36 |
|  | LTR37 | LTR37A, LTR37B |
|  | LTR48 | LTR48, LTR48B |
|  | LTR51 | LTR51 |
|  | LTR54 | LTR54, LTR54B |

| **Superfamily** | **Family** | **Name/Group** |
| --- | --- | --- |
| **1. ERV1 (cont.)** | LTR58 | LTR58 |
|  | LTR59 | LTR59 |
|  | LTR60 | LTR60 |
|  | LTR61 | LTR61 |
|  | LTR64 | LTR64 |
|  | LTR65 | LTR65 |
|  | LTR68 | LTR68 |
|  | LTR72 | LTR72, LTR72B |
|  | LTR75_1 | LTR75_1 |
|  | LTR76 | LTR76 |
|  | LTR77 | LTR77 |
|  | LTR78 | LTR78, LTR78B |
|  | MER101 | MER101, MER101B |
|  | MER110 | MER110, MER110A |
|  | MER31 | MER31, MER31A, MER31B |
|  | MER34 | MER34, MER34A, MER34A1, MER34B, MER34C, MER34C2, MER34D |
|  | MER39 | MER39, MER39B |
|  | MER4 | MER4, MER4A, MER4A1, MER4B, MER4C, MER4D, MER4D0, MER4D1, MER4E, MER4E1 |
|  | MER41 | MER41, MER41A, MER41B, MER41C, MER41D, MER41E, MER41G |
|  | MER49 | MER49 |
|  | MER50 | MER50, MER50B, MER50C |
|  | MER51 | MER51, MER51A, MER51B, MER51C, MER51D, MER51E |
|  | MER52 | MER52, MER52A, MER52C, MER52D |
|  | MER57 | MER57, MER57A, MER57A1, MER57B1, MER57B2, MER57C1, MER57C2, MER57D, MER57E1, MER57E2, MER57E3, MER57F |
|  | MER61 | MER61, MER61A, MER61B, MER61C, MER61D, MER61E, MER61F |
|  | MER65 | MER65, MER65A, MER65B, MER65C, MER65D |
|  | MER66 | LTR73, MER66, MER66A, MER66B, MER66C, MER66D |
|  | MER67 | MER67A, MER67B, MER67C, MER67D |
|  | MER72 | MER72, MER72B |
|  | MER83 | MER83, MER83A, MER83B, MER83C |
|  | MER84 | MER84 |
|  | MER87 | MER87, MER87B |
|  | MER89 | MER89 |
|  | MER90 | MER90a |
|  | MER92 | MER92A, MER92B |
|  | PAB | PABL_A, PABL_B |
|  | PRIMA4 | PRIMA4, PRIMAX |
|  | PRIMA41 | PRIMA41 |
|  | PrimLTR79 | PrimLTR79 |
|  |  |  |
| **Superfamily** | **Family** | **Name/Group** |
| **2. ERVK** | HERVK10/  HERVK (HML-2) | LTR5, LTR5_Hs, LTR5A, LTR5B, HERVK |
|  | HERVK14/  HERVK (HML-1) | HERVK14, HERVK14C, LTR14, LTR14A, LTR14B, LTR14C |
|  | HERVK9/  HERVK (HML-3) | HERVK9, MER9a1, MER9a2, MER9a3, MER9B |
|  | HERVK13/  HERVK (HML-4) | HERVK13, LTR13, LTR13A |
|  | HERVK22/  HERVK (HML-5) | HERVK22, LTR22, LTR22A, LTR22B, LTR22C |
|  | HERVK3/  HERVK (HML-6) | HERVK3, LTR3, LTR3A, LTR3B |
|  | HERVK11D/  HERVK (HML-7) | HERVK11D, MER11D |
|  | HERVK11/  HERVK (HML-8) | HERVK11, MER11A, MER11B, MER11C |
|  | HERVKC4/  HERVK (HML-10) | HERVKC4 |
| **3. ERVL** | ERV3-16A3 | ERV3-16A3 |
|  | ERVL | ERVL, ERVLB4, ERVLE, HERVL, MLT2A1, MLT2A2, MLT2B1, MLT2B2, MLT2B3, MLT2B4, MLT2B5, MLT2C1, MLT2C2, MLT2D, MLT2E, MLT2F |
|  | HERV16 | HERV16, LTR16A, LTR16A1, LTR16A2, LTR16B, LTR16B1, LTR16B2, LTR16C, LTR16D, LTR16D1, LTR16D2, LTR16E1, LRE16E2 |
|  | HERV18 | HERVL18, LTR18A, LTR18B |
|  | HERV32 | HERVL32, LTR32 |
|  | HERV47 | LTR47A, LTR47B |
|  | HERVL33 | LTR33, LTR33A, LTR33B, LTR33C, LTR41, LTR41B |
|  | HERVL40 | HERVL40, LTR40a, LTR40A1, LTR40b, LTR40c |
|  | HERVL42 | LTR42 |
|  | HERVL50 | LTR50 |
|  | HERVL52 | LTR52 |
|  | HERVL53 | LTR53, MER88 |
|  | HERVL54 | MER54A, MER54B |
|  | HERVL57 | LTR57 |
|  | HERVL66 | HERVL66, LTR66 |
|  | HERVL67 | LTR67B |
|  | HERVL68 | MER68, MER68B |
|  | HERVL69 | LTR69 |
|  | HERVL70 | MER70, MER70A, MER70B, MER70C |
|  | HERVL73 | MER73 |
|  | HERVL74 | HERVL74, MER74A, MER74B, MER74C |
|  | HERVL75 | LTR75, LTR75B |
|  | LTR62 | LTR62 |
|  | LTR79 | LTR79 |
|  | LTR80 | LTR80A, LTR80B |
|  | LTR82 | LTR82A, LTR82B |
|  | LTR83 | LTR83 |

| **Superfamily** | **Family** | **Name/Group** |
| --- | --- | --- |
| **3. ERVL (cont.)** | LTR84 | LTR84a, LTR84b |
|  | LTR86 | LTR86A1, LTR86A2, LTR86B1, LTR86B2, LTR86C |
|  | MER21 | MER21, MER21A, MER21B, MER21C |
|  | MER76 | MER76 |
|  | MER77 | MER77, MER77B |
| **4. ERVL-MaLR** | MLT1 | MLT1, MLT1A, MLT1A0, MLT1A1, MLT1B, MLT1C, MLT1D, MLT1E, MLT1E1, MLT1E1A, MLT1E2, MLT1E3, MLT1F, MLT1F1, MLT1F2, MLT1G, MLT1G1, MLT1G3, MLT1H, MLT1H1, MLT1H2, MLT1I, MLT1J, MLT1J1, MLT1J2, MLT1K, MLT1L, MLT1M, MLT1N2 |
|  | MST | MST, MSTA, MSTB, MSTB1, MSTB2, MSTC, MSTD |
|  | THE1 | MLT, THE1, THE1A, THE1B, THE1C, THE1D |
