# Supplementary material for: EnHERV: Enrichment analysis of specific human endogenous retrovirus patterns and their neighboring genes
Source: PLoS One. 2017 May 4;12(5):e0177119. doi: 10.1371/journal.pone.0177119 (PMC5417679; doi:10.1371/journal.pone.0177119)
Supplement: S5 Table — (DOCX) [file pone.0177119.s007.docx]

| HERV regulator | HERV name | Gene name | Reference |
| --- | --- | --- | --- |
| 1. Enhancer | ERV9 LTR | β-globin locus | [1] |
|  | HERV-E | Amy1 (salivary amylase) | [2] |
| 2. Promoter | HERV-L LTR | β1,3-galactosyltransferase 5 | [3] |
|  | HERV-E | APOCI (apolipoprotein CI) | [4] |
|  | HERV-H LTR | DSCR4 and DSCR8 (Down syndrome critical region) | [5] |
|  | HERV-E | EDNRB (endothelin receptor B) | [4] |
|  | HERV-H | NAIP (neuronal apoptosis inhibitory protein) | [2] |
|  | ERV9 LTR | ZNF80 (zinc finger protein) | [6] |
| 3.Polyadenylation signals | HERV-K (KML2) LTR | LEPR (human leptin receptor) | [2] |
| 4. Splice sites | HERV-H | PLA2L (phospholipase A2-like) | [7] |
| 5. Antisense regulators | LTR91 | CEBZ | [8] |

**S5 Table** Evidences of LTR involved in gene expression

1. Long, Q., et al., *A Long Terminal Repeat of the Human Endogenous Retrovirus ERV-9 Is Located in the 5' Boundary Area of the Human [beta]-Globin Locus Control Region.* Genomics, 1998. 54(3): p. 542-555.

2. Jern, P. and J.M. Coffin, *Effects of Retroviruses on Host Genome Function.* Annual Review of Genetics, 2008. 42(1): p. 709-732.

3. Dunn, C.A., P. Medstrand, and D.L. Mager, *An endogenous retroviral long terminal repeat is the dominant promoter for human Beta1,3-galactosyltransferase 5 in the colon.* Proceedings of the National Academy of Sciences of the United States of America, 2003. 100(22): p. 12841-12846.

4. Medstrand, P., J.-R. Landry, and D.L. Mager, *Long Terminal Repeats Are Used as Alternative Promoters for the Endothelin B Receptor and Apolipoprotein C-I Genes in Humans.* Journal of Biological Chemistry, 2001. 276(3): p. 1896-1903.

5. Dunn, C.A., et al., *Transcription of two human genes from a bidirectional endogenous retrovirus promoter.* Gene, 2006. 366(2): p. 335-342.

6. Di Cristofano, A., et al., *Characterization and genomic mapping of the ZNF80 locus: expression of this zinc-finger gene is driven by a solitary LTR of ERV9 endogenous retrovrial family.* Nucleic Acids Research, 1995. 23(15): p. 2823-2830.

7. Feuchter-Murthy, A.E., J.D. Freeman, and D.L. Mager, *Splicing of a human endogenous retrovirus to a novel phospholipase A2 related gene.* Nucleic Acids Research, 1993. 21(1): p. 135-143.

8. Buzdin, A., *Human-Specific Endogenous Retroviruses.* TheScientificWorldJOURNAL, 2007. 7: p. 1848-1868.
